# Evaluation of a Novel Multiplex PCR Assay for Vesicular Viruses

**DOI:** 10.3390/ijms27104477

**Published:** 2026-05-16

**Authors:** Giuseppe Sberna, Maria Beatrice Valli, Francesca Colavita, Fabiano Brillo, Gabriella Rozera, Claudia Minosse, Fabrizio Maggi, Eleonora Lalle

**Affiliations:** Laboratory of Virology and Laboratories of Biosafety, National Institute for Infectious Diseases Lazzaro Spallanzani—IRCCS, 00149 Rome, Italy

**Keywords:** multiplex PCR, MPXV, HSV-1, HSV-2, VZV, Novaplex, monkeypox virus, herpes simplex virus, varicella zoster virus

## Abstract

Differentiation and detection of viruses causing vesicular/mucocutaneous lesions are essential for patient management. This study evaluated the analytical and clinical performance of the Novaplex™ HSV-1&2/VZV/MPXV multiplex real-time PCR assay (Novaplex), designed for the simultaneous detection of monkeypox virus (MPXV), herpes virus types 1 and 2 (HSV), and varicella-zoster virus (VZV). It was compared with Singleplex PCRs as reference methods. Analytical sensitivity was assessed only for MPXV using viral stocks of clades Ia, Ib, and IIb, while clinical performances for HSV, VZV and MPXV were evaluated on 93 residual clinical samples. Novaplex discriminated clade II from clade I viruses and demonstrated low limits of detection for MPXV (clade Ia: 2.9 Log TCID_50_/mL; clade Ib: 1.9 Log TCID_50_/mL; clade IIb: 1.4 Log TCID_50_/mL). Clinically, the assay showed high overall sensitivity (97.1%) and specificity (100%), with almost perfect agreement with Singleplex PCR (κ = 0.947). Stratified results by viruses: HSV showed κ = 1.000 and 100% of sensitivity, MPXV showed a sensitivity of 97.8% with κ = 0.969, and VZV showed a κ = 0.914 with a sensitivity of 87.5%. Specificity was 100% for all three viruses. Novaplex offers a robust, efficient diagnostic approach for simultaneous detection of MPXV, HSV-1/2, and VZV, supporting timely clinical decision making and enhanced outbreak preparedness.

## 1. Introduction

Infections caused by herpes viruses and orthopoxviruses represent a significant diagnostic challenge for healthcare systems, due to the similarities in clinical manifestations and the possible overlap of symptoms [1,2,3]. Herpes simplex virus type 1 (HSV-1) and type 2 (HSV-2) in higher-income countries are among the most common causative agents of mucocutaneous lesions [4], while varicella-zoster virus (VZV) is responsible for both primary chickenpox and reactivation in the form of herpes zoster [5,6]. Both viruses have the ability to disseminate to the central nervous system (CNS) after reactivation from lifelong latency in sensory ganglia, causing severe viral meningoencephalitis. These herpetic viruses are among the leading causes of viral meningoencephalitis in immunocompetent adults [7].

After spreading in non-endemic areas since 2022, the multi-country reports of active transmission of both clades of monkeypox virus (MPXV) have highlighted the urgent need for rapid and accurate diagnostic assays to distinguish among pathogens causing vesicular rash with similar clinical presentations, particularly in the early stages of infection [8,9,10].

In this context, molecular biology techniques, and in particular assays based on multiplex real-time PCR, have taken on a central role in virological diagnosis thanks to their high sensitivity, specificity, and ability to simultaneously detect multiple pathogens in a single reaction [11,12]. The use of standardized commercial kits offers additional advantages, including reduced inter-laboratory variability, faster turnaround times, and easier integration into diagnostic workflows. However, the routine adoption of such multiplex systems requires rigorous evaluation of their analytical and clinical performance to ensure their reliability in different epidemiological contexts and different types of biological samples.

The evaluation of analytical performance includes fundamental parameters such as detection limit, precision, reproducibility, and analytical specificity. At the same time, clinical performance analysis allows the determination of diagnostic sensitivity and specificity compared to reference methods. These aspects are particularly relevant for pathogens that require timely identification in order to allow proper therapeutic treatment and to undertake prompt public health measures [13,14].

The aim of this study was to evaluate the analytical and clinical sensitivity of the commercial multiplex assay Novaplex™ HSV-1&2/VZV/MPXV (Novaplex), with particular focus on its performance in detecting MPXV compared with a reference Singleplex PCR assay for orthopoxvirus. In addition, we assessed its ability to simultaneously identify HSV-1, HSV-2, and VZV, highlighting its potential role in the differential diagnosis of viral skin and mucosal lesions. By comparing the multiplex assay with established Singleplex PCR methods, we aimed to determine its diagnostic accuracy and explore its potential contribution to streamlining diagnostic workflows and improving timely and appropriate patient management.

## 2. Results

### 2.1. Analytical Sensitivity

**Figure 1:**
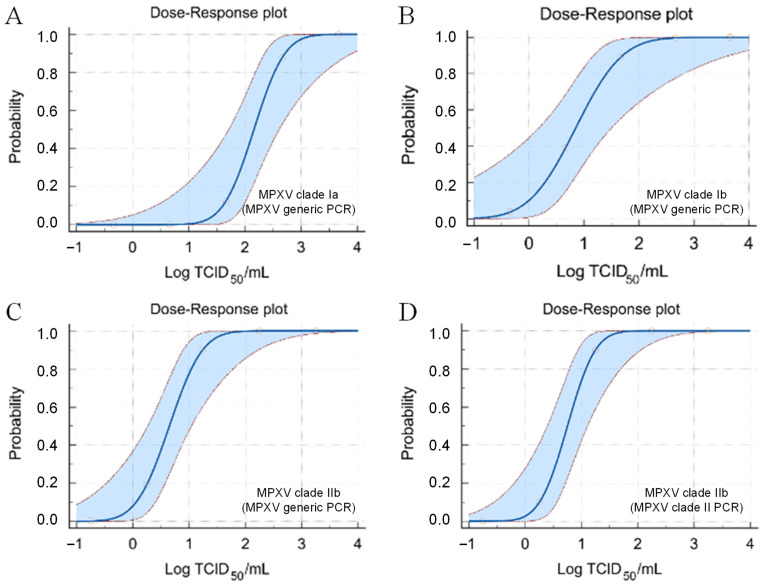
Probit analysis for the Novaplex™ HSV-1&2/VZV/MPXV assay considering the MPXV PCR: (**A**) Probit analysis for MPXV clade Ia considering the MPXV generic PCR. (**B**) Probit analysis for MPXV clade Ib considering the MPXV generic PCR. (**C**) Probit analysis for MPXV clade IIb considering the MPXV generic PCR. (**D**) Probit analysis for MPXV clade IIb considering the MPXV clade II PCR.

The analytical sensitivity of the Novaplex assay was evaluated using the viral stocks available: clades Ia, Ib, and IIb of MPXV. Considering the Novaplex generic MPXV PCR assay, the LoD for clade Ia was 2.9 (95% CI: 2.5–4.3) Log TCID_50_/mL (LoD: 3.5 Log copies/mL; Table 1; Figure 1A), for clade Ib it was 1.9 (95% CI: 1.4–4.4) Log TCID_50_/mL (LoD: 2.9 Log copies/mL; Table 1; Figure 1B), and for clade IIb it was 1.4 (95% CI: 1.0–2.6) Log TCID_50_/mL (LoD: 2.9 Log copies/mL; Table 1; Figure 1C).

In contrast, when considering the clade II-specific MPXV PCR assay, all dilutions of the clade Ia and Ib viral stocks tested negative, making it impossible to calculate a LoD. For the clade IIb viral stock, a LoD of 1.4 (95% CI: 1.1–2.4) Log TCID_50_/mL was obtained (LoD: 2.8 Log copies/mL; Table 1; Figure 1D).

### 2.2. Clinical Sensitivity and Specificity

Using the Singleplex system, 25 samples tested negative for all viral pathogens included in the Novaplex assay and 68 tested positive for at least one of them. Among the latter, 46 samples were positive for MPXV, 13 for HSV-1 or HSV-2, 1 coinfected with HSV-1 and HSV-2, and 8 for VZV. All 93 samples were subsequently tested with the Novaplex assay, yielding 27 negative and 67 positive results. Specifically, 45 samples were positive for MPXV, 13 for HSV-1 or HSV-2, 1 coinfected with HSV-1 and HSV-2 (this sample was counted twice for analytical purposes: once as HSV-1–positive and once as HSV-2–positive), and 7 for VZV. Based on these results, the clinical sensitivity and specificity of the Novaplex assay were calculated, along with its agreement with the reference method.

Across all samples analyzed, the Novaplex assay demonstrated a high diagnostic performance with an overall sensitivity of 97.1% (95% CI: 89.9–99.7%), an overall specificity of 100.0% (95% CI: 86.3–100.0%), and an almost perfect agreement with Singleplex PCR assays (κ = 0.947; 95% CI: 0.874–1.000; Table 2A).

More in detail, when stratified by virus, the results remained comparable, with almost perfect agreement observed for all pathogens. Specifically, Cohen’s kappa was 0.914 (95% CI: 0.748–1.000) for VZV, 0.969 (95% CI: 0.910–1.000) for MPXV, and 1.000 (95% CI: 1.000–1.000) for HSV (Table 2B, Table 2C and Table 2D, respectively). For HSV, Novaplex demonstrated a sensitivity of 100.0% (95% CI: 78.2–100.0%) and a specificity of 100.0% (95% CI: 86.3–100.0%; Table 2D); for VZV, a sensitivity of 87.5% (95% CI: 47.4–99.7%) and a specificity of 100.0% (95% CI: 86.3–100.0%; Table 2B); and for MPXV, a sensitivity of 97.8% (95% CI: 88.5–99.9%) and a specificity of 100.0% (95% CI: 86.3–100.0%; Table 2C).

**Figure 2:**
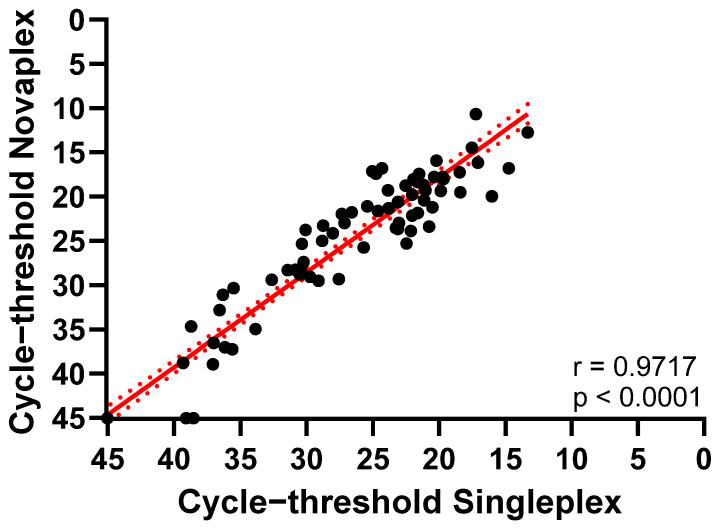
Linear regression analysis between Singleplex PCR assay and the Novaplex™ HSV-1&2/VZV/MPXV assay.

In addition, Ct values between Novaplex and Singleplex were compared using correlation and linear regression analysis obtaining *p* < 0.0001 and r = 0.9717 (Figure 2).

Two samples that tested negative with Novaplex but positive with Singleplex showed Ct values of 38.5 (MPXV-positive) and 39.1 (VZV-positive).

Moreover, it should be noted that Novaplex, being a multiplex system, was able to detect HSV-1 in five samples tested with a specific Singleplex PCR different from HSV (Table 3).

Finally, to verify the absence of cross-reactivity in the Novaplex, five samples that had tested positive for Epstein–Barr virus (EBV) using a Singleplex assay and five samples that had tested positive for Cytomegalovirus (CMV) were evaluated. Despite exhibiting Ct values ranging from 27 to 32 with Singleplex, all samples resulted negative with the Novaplex assay.

## 3. Discussion

In modern society, there is increasing pressure to find solutions quickly, especially in clinical settings where rapid diagnosis can save lives or may be crucial for healthcare management. To this end, multiplex PCRs have become essential for rapid diagnosis, reducing hands-on time, reagent consumption, and overall turnaround time without compromising analytical rigor. These operational efficiencies are particularly impactful in syndromic presentations (i.e., vesicular or mucocutaneous lesions) where rapid, parallel interrogation of plausible etiologies improves diagnostic yield and accelerates clinical decision making. This need is further emphasized by the recent epidemiology of mpox. The multi-country outbreak of MPXV that began in 2022 was characterized by unprecedented human-to-human transmission [3,15,16]. Lesions were predominantly localized in the genital region rather than the face, palms, and soles, as historically reported in Central and West Africa, and the affected population was primarily men who have sex with men (MSM), many of whom had a high prevalence of sexually transmitted infections (STIs), including herpesviruses [17,18,19]. These observations highlight the need for integrated sexual health services and targeted prevention strategies, including routine STIs screening, to limit both MPXV spread and coexisting infections in at-risk communities.

For these reasons, we examined the Novaplex™ HSV-1&2/VZV/MPXV multiplex assay by comparing it to single PCRs. Novaplex demonstrated an almost perfect agreement with Singleplex PCR, both when considering all samples (κ = 0.947) and when divided according to the pathogen under examination: κ = 1.000 for HSV 1/2, κ = 0.969 for MPXV, and κ = 0.914 for VZV. Discordant clinical results occurred at high Ct values, amplification signals observed near the respective limits of detection. At these Ct ranges, stochastic amplification effects, minimal viral nucleic acid input, and pre-analytical variability are known to contribute to reduced reproducibility, regardless of the testing format employed [20,21,22].

Sensitivity and specificity were similarly high for each pathogen, confirming the assay’s robustness in detecting viral infections that often present with overlapping vesicular or mucocutaneous lesions. Furthermore, no cross-reactivity of the Novaplex was observed with other viruses potentially associated with vesicular lesions, including EBV and CMV.

Only two samples with very low viral DNA loads, detected by the Singleplex assays at Ct values near the detection limits of both methods, were missed by the Novaplex™ assay. These discordant results observed near the limit of detection are probably due to a limitation of PCR technologies and should not be attributed to one platform over another. On the other hand, since a multiplex approach can detect multiple pathogens simultaneously, Novaplex allowed us to identify HSV-1 in five samples in which singleplex PCR specific for this pathogen had not been requested and not tested in this study. Probably, these positives would likely have remained undiagnosed following a single-target approach. This result highlights three key advantages of multiplexing: greater diagnostic yield, as it expands the etiological spectrum investigated in a non-hypothesis-dependent manner and intercepts co-infections or alternative causes not suspected a priori; process efficiency, with reduced response times and reagent/sample consumption; and direct clinical impact, because the timely identification of HSV-1 in the five samples potentially anticipated therapeutic decisions and control measures, improving patient management. Notably, the use of real-world diagnostic samples reflects routine laboratory conditions and supports the relevance of the reported clinical performance.

Furthermore, Novaplex demonstrated excellent low LoDs for MPXV detection. Using the generic MPXV PCR, the LoDs were 2.9 Log TCID_50_/mL for clade Ia, 1.9 Log TCID_50_/mL for clade Ib, and 1.4 Log TCID_50_/mL for clade IIb. With the clade II-specific PCR of the Novaplex, all dilutions of clade Ia and clade Ib tested negative (precluding LoD estimation), while clade IIb resulted in an LoD of 1.4 Log TCID_50_/mL, thus supporting the clade-specificity of the methods.

It should be noted that Novaplex follows contemporary guidelines for mpox [16], emphasizing the need for assays that target conserved orthopox/MPXV regions [23] and, where feasible, clade-informative analytes to mitigate gene target failure as the virus evolves, reinforcing the value of multiplex panels that combine generic and clade-specific targets with differential vesicular diagnosis. It should also be noted that the WHO Disease Outbreak News reported a recombinant MPXV with genomic elements of clades Ib and IIb in travel-associated cases, underscoring the need to empirically verify detection and classification of recombinant lineages by both systems used here (Novaplex multiplex and Singleplex reference), including assessments of inclusivity, potential target drop outs, and interpretive rules when generic and clade specific channels diverge [3,16].

This study presents some limitations. First, the number of VZV-positive and HSV-positive clinical samples was limited; this could prevent complete evaluation of assay performance, particularly relating to sensitivity for these viruses and their ranges. Second, we lacked VZV and HSV viral stocks, preventing analytical sensitivity testing for these viruses analogous to our MPXV analyses. Third, this was a single-center study using primarily lesion and respiratory swabs; performances in other matrices and inter-laboratory reproducibility were not assessed. Moreover, samples analyzed in this study are residual clinical specimens, with limited volume. For this reason, it was not possible to verify the discrepancies observed between the compared methods using an additional, independent method.

## 4. Materials and Methods

### 4.1. Viruses

The following viral isolates were used to assess the analytical sensitivity of the Novaplex™ HSV-1&2/VZV/MPXV: clade IIb, hMpxV/Italy/un-INMI-Pt2/2022 (GISAID: EPI_ISL_13251120, GenBank: ON745215.1), obtained from a patient hospitalized at INMI in 2022; and clade Ia and clade Ib, both provided by WHO BioHub Facility (catalog reference 2023-WHO-LS-008 and 2024-WHO-LS-003, respectively) and originating from the Democratic Republic of the Congo (WHO BioHub Facility, Spiez, Switzerland). To prepare viral stocks, Vero E6 cells, maintained in Minimum essential Medium supplemented with 1% Penicillin/1% Glutammin and 10% Fetal Bovine Serum, at 37 °C in a 5% CO_2_ atmosphere, were infected in the Biosafety Level 3 (BSL3) facility with the MPXV isolates. After being frozen and thawed three times, cell lysates were cleared, aliquoted, and stored at −80 °C. Viral stocks titers were obtained by limiting dilution assay on the Vero E6 cells and expressed as TCID_50_/mL, according to the Reed and Muench method [24]. In addition, to quantify the copies/mL of MPXV DNA in the viral stocks the Bio-Rad QX200 AutoDG Digital Droplet PCR system (Bio-Rad [25]) was used as previously described [26]. Briefly, 0.9 μM of primers and 0.25 μM of probe were added to ddPCR Supermix (Bio-Rad Laboratories, Hercules, CA, USA [25]). To maintain consistent quantification, DNA from each sample was processed in three separate wells, and the results were combined during analysis. Following the PCR reaction, the droplets were read using a QX100 droplet reader, and the data were analyzed with QuantaSoft software version 1.7.4.0917 (Bio-Rad Laboratories [25]).

### 4.2. Clinical Samples

A total of 93 clinical samples (13 residual nasopharyngeal swabs and 80 residual skin lesion swabs) collected from patients presenting at INMI for MPXV diagnosis or other vesicular viruses were used to evaluate the clinical performance of the Novaplex. After laboratory diagnosis, specimens were stored at −80 °C until the day of analysis with the Novaplex. In this study, specimens were selected based on the availability of residual sample volume among positive and negative samples tested for routine activities. In addition, five EBV-positive samples and five CMV-positive samples were used to carry out cross-reactivity analyses.

The molecular diagnosis of clinical samples was performed using a commercial Singleplex assay (Singleplex; Altona Diagnostics Italia S.r.l., Segrate, Italy [27]). The assay is based on an automated platform (AltoStar, Altona Diagnostics Italia S.r.l., Segrate, Italy [27]) which integrates nucleic acid extraction and plate-based PCR setup followed by a real-time PCR target-specific by using the Bio-Rad CFX96 thermal cycler (Bio-Rad Laboratories [25]).

### 4.3. Novaplex™ HSV-1&2/VZV/MPXV Assay

Novaplex assay is able to detect simultaneously 4 targets: HSV-1, HSV-2, VZV and MPXV. Regarding MPXV, the test amplified two different regions of the genome, with two different probes: one able to identify both MPXV clade I and II and the second targeting only clade II. Nucleic acids were extracted using the automated Nimbus system (Seegene Italy, Genova, Italy [28]). Real-time PCR analysis was carried out using the CFX96 thermal cycler (Bio-Rad Laboratories, Hercules, CA, USA [25]). Data analysis was conducted automatically with Seegene Viewer software (version 2.0) [28]. All procedures were carried out following the manufacturer’s instructions.

### 4.4. Statistical Analysis

Cohen’s kappa was calculated using GraphPad QuickCalcs (GraphPad Software [29]), while sensitivity and specificity were determined with MedCalc Online statistical calculators (MedCalc Software [30]). Probit regression analysis was performed to evaluate the limit of detection (LoD) for MPXV at the 95% confidence interval (95% CI) by using MedCalc statistical software version 23.5.0 (MedCalc Software [30]). Linear regression and correlation analysis were used to compare cycle-threshold (Ct) values between the two systems using GraphPad Prism software version 10.6.1 (GraphPad Software, La Jolla, CA, USA [29]). For analysis and graphical representation an arbitrary value of 45.01 was assigned to negative results.

## 5. Conclusions

This study demonstrates that the Novaplex™ HSV-1&2/VZV/MPXV assay represents a robust and clinically valuable diagnostic tool, capable of integrating rapid detection, high analytical performance, and an efficient solution for the simultaneous detection of vesicle-associated viruses, thereby supporting more efficient patient management and reinforcing preparedness in the face of evolving viral epidemiology.

## Figures and Tables

**Table 1:** Analytical sensitivity of the Novaplex™ HSV-1&2/VZV/MPXV assay considering the MPXV PCR: (**A**) results from the generic MPXV PCR; (**B**) results from the clade II-specific assay. The clade II-specific PCR yields no amplification for clade Ia and Ib samples, precluding the limit of detection (LoD) determination, whereas clade IIb dilutions are detectable and allow LoD estimation. TCID_50_/mL: 50% tissue culture infectious dose/mL.

**A**	**MPXV Generic PCR**							
MPXV Clade Ia			MPXV Clade Ib			MPXV Clade IIb		
TCID_50_/mL	copies/mL	Novaplex PCR (Positive/Total)	TCID_50_/mL	copies/mL	Novaplex PCR (Positive/Total)	TCID_50_/mL	copies/mL	Novaplex PCR (Positive/Total)
10^3.66^	10^4.28^	8/8	10^3.66^	10^4.66^	6/6	10^3.25^	10^4.70^	10/10
10^2.66^	10^3.28^	7/8	10^2.66^	10^3.66^	6/6	10^2.25^	10^3.70^	10/10
10^1.66^	10^2.28^	1/8	10^1.66^	10^2.66^	5/6	10^1.25^	10^2.70^	9/10
10^0.66^	10^1.28^	0/8	10^0.66^	10^1.66^	3/6	10^0.25^	10^1.70^	2/10
/	/	/	10^−0.34^	10^0.66^	0/6	10^−0.75^	10^0.70^	0/10
**B**	**MPXV Clade II PCR**							
MPXV Clade Ia			MPXV Clade Ib			MPXV Clade IIb		
TCID_50_/mL	copies/mL	Novaplex PCR (Positive/Total)	TCID_50_/mL	copies/mL	Novaplex PCR (Positive/Total)	TCID_50_/mL	copies/mL	Novaplex PCR (Positive/Total)
10^3.66^	10^4.28^	0/8	10^3.66^	10^4.66^	0/6	10^3.25^	10^4.70^	10/10
10^2.66^	10^3.28^	0/8	10^2.66^	10^3.66^	0/6	10^2.25^	10^3.70^	10/10
10^1.66^	10^2.28^	0/8	10^1.66^	10^2.66^	0/6	10^1.25^	10^2.70^	9/10
10^0.66^	10^1.28^	0/8	10^0.66^	10^1.66^	0/6	10^0.25^	10^1.70^	1/10
/	/	/	10^−0.34^	10^0.66^	0/6	10^−0.75^	10^0.70^	0/10

**Table 2:** Comparison between Singleplex PCR assay and the Novaplex™ HSV-1&2/VZV/MPXV assay. (**A**) Comparison between Singleplex and the Novaplex considering all samples. (**B**) Comparison between Singleplex and Novaplex considering only VZV. (**C**) Comparison between Singleplex and Novaplex considering only MPXV. (**D**) Comparison between Singleplex and Novaplex considering only HSV.

A		Singleplex			B		Singleplex VZV		
		Positive	Negative	Total			Positive	Negative	Total
**Novaplex**	**Positive**	67	0	67	**Novaplex VZV**	**Positive**	7	0	7
	**Negative**	2	25	27		**Negative**	1	25	26
	**Total**	69	25	94		**Total**	8	25	33
**C**		**Singleplex MPXV**			**D**		**Singleplex HSV**		
		**Positive**	**Negative**	**Total**			**Positive**	**Negative**	**Total**
**Novaplex** **MPXV**	**Positive**	45	0	45	**Novaplex HSV**	**Positive**	15	0	15
	**Negative**	1	25	26		**Negative**	0	25	25
	**Total**	46	25	71		**Total**	15	25	40

**Table 3:** HSV-1 detection by Novaplex™ HSV-1&2/VZV/MPXV assay in samples on which specific Singleplex PCR was not performed.

Singleplex		Novaplex				
Target	Results (Ct)	MPXV Generic (Ct)	MPXV Clade II (Ct)	HSV-1 (Ct)	HSV-2 (Ct)	VZV (Ct)
MPXV	Positive (27.1)	Positive (23.1)	Positive (22.9)	Positive (38.4)	Negative (45.01)	Negative (45.01)
MPXV	Negative (45.01)	Negative (45.01)	Negative (45.01)	Positive (16.6)	Negative (45.01)	Negative (45.01)
MPXV	Positive (27.5)	Positive (30.6)	Positive (29.3)	Positive (34.5)	Negative (45.01)	Negative (45.01)
MPXV	Positive (19.7)	Positive (17.1)	Positive (17.8)	Positive (35.0)	Negative (45.01)	Negative (45.01)
VZV	Positive (13.3)	Negative (45.01)	Negative (45.01)	Positive (39.8)	Negative (45.01)	Positive (12.7)

## Data Availability

The original contributions presented in the study are included in the article; further inquiries can be directed to the corresponding author.
